# Chemical Composition of *Mentha pulegium* and its Contributions to Antimicrobial, Antioxidant, and Insecticidal Activities: Insights from In Vitro and Molecular Docking Studies

**DOI:** 10.1021/acsomega.5c04188

**Published:** 2025-10-01

**Authors:** Nacira Amara, Mohamed Kouider Amar, Nabil Touzout, Houda Saoud, Nadjet Boucheman, Madina Benzineb, Muhammad Farhan Saeed, Jakub Černý, Aftab Jamal

**Affiliations:** † Département de Biologie, Faculté des Sciences de la Nature et de la Vie, 213442 Université Blida 1, BP 270, Blida 09000, Algeria; ‡ Laboratory of Quality Control, Physicochemical Department, 474411 SAIDAL of Medea, Medea 26000, Algeria; § Department of Agronomy, Faculty of Sciences, Pole Urban Ouzera, University of Dr. Yahia Fares of Medea, Medea 26000, Algeria; ∥ Laboratoire des Molécules Bioactives et leurs Applications, Département de Biologie Appliquée, Faculté des Sciences Exactes et des Sciences de la Nature et de la Vie, 257235 Université Echahid Cheikh Larbi Tebessi, Tebessa 12000, Algeria; ⊥ Department of Environmental Sciences, COMSATS University Islamabad, Vehari Campus, Vehari 61100, Pakistan; # Department of Silviculture, Faculty of Forestry and Wood Technology, Mendel University in Brno, Zemědělská 3, Brno 613 00, Czech Republic; ¶ Department of Soil and Environmental Sciences, Faculty of Crop Production Sciences, 66936 The University of Agriculture, Peshawar 25130, Pakistan

## Abstract

This study investigated the potential of essential oils (EOs) derived from *Mentha pulegium* in two Algerian regions, Attatba and Hammam Melouane, as sources of bioactive compounds with antimicrobial, antioxidant, and insecticidal properties. Botanical surveys confirmed the presence of three *Mentha* species (*Mentha spicata*, *M. pulegium*, and *Mentha rotundifolia*) in Attatba, along with a fourth species (*Mentha suaveolens*) in Hammam Melouane. Gas chromatography–mass spectrometry (GC–MS) analysis revealed distinct chemotypes: the EO from Hammam Melouane presented a pulegone-dominant profile (61%), whereas the EO from Attatba presented (+)-limonene/piperitone/piperitenone chemotypes of 41.99%, 23.08%, and 12.06%, respectively. The antioxidant potential of *M. pulegium* EOs from both sites was assessed and found to be weak. Insecticidal assays against *Aphis spiraecola* demonstrated that both formulations had mortality rates ranging from 31.34 ± 1.7% to 81.04 ± 1.78% and from 45.17 ± 0.88% to 94.4 ± 1.10%, respectively. The LD_50_ values against *A. spiraecola* were determined to be 107.6 μL/mL for Hammam Melouane and 142.3 μL/mL for Attatba. Furthermore, molecular docking simulations revealed that piperitenone is a significant multitarget compound, with high binding affinities of −6.4 kcal/mol for DNA gyrase (1KZN), −5.3 kcal/mol for human peroxiredoxin (1HD2), and −7.4 kcal/mol for acetylcholinesterase (4EY7). This study confirms the insecticidal properties of *M. pulegium* EOs from the Attatba and Hammam Melouane regions, underscoring the potential of *M. pulegium* EO in the discovery of new therapeutic agents and highlighting its significance for future applications.

## Graphical abstract

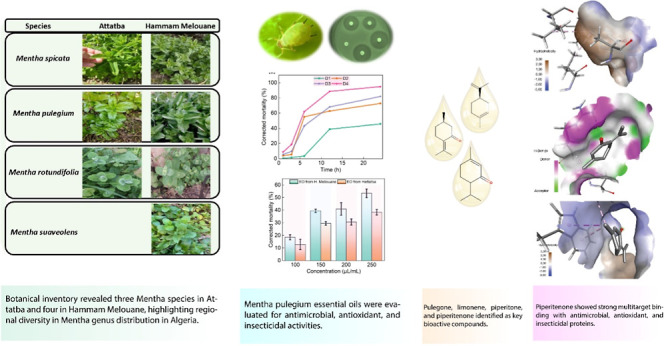

## 1 Introduction

Aromatic and medicinal plants are invaluable reservoirs of bioactive molecules capable of synthesizing a diverse array of natural compounds.^1,2^ These plants are rich in chemical constituents that endow them with potent antioxidant, antimicrobial, and insecticidal properties. Extensive research on various aromatic and medicinal plants has led to the development of natural antioxidant formulations, which have demonstrated their applicability across multiple industries, including food, cosmetics, and pharmaceuticals.^3^ The antimicrobial compounds found in these plants are of particular interest, as multidrug-resistant bacteria pose an increasing global health concern, especially in foodborne infections and nosocomial contaminations.^4,5^ In this context, EOs are promising alternatives to synthetic chemicals because of their environmental safety, broad-spectrum efficacy against pests, and diverse mechanisms of action.^6^

The Mentha species, which belong to the *Lamiaceae* family, include rapidly growing aromatic herbaceous plants with remarkable ecological adaptability. Due to their resilience, *Mentha* species are cultivated under various climatic conditions across different regions of the world, including Europe, Asia, Africa, Australia, and North America.^7–9^ Their rich composition of bioactive compounds, particularly monoterpenoids and polyphenols, has led to extensive utilization in traditional medicine and as culinary herbs.

Among these species, *Mentha pulegium L*., commonly known as pennyroyal, is a perennial herb belonging to the Lamiaceae family.^10^ Native to North Africa, Europe, Asia Minor, and the Middle East, it thrives in the wild, particularly in humid plains and mountainous regions, and has a broad geographical distribution worldwide.^11^ In Algeria, it is widely recognized under the local name “Fliou” and is among the most frequently used medicinal plants. Its EO and aerial parts have long been employed in traditional medicine, particularly for treating digestive disorders such as dyspepsia and intestinal colic. Additionally, *M. pulegium* holds significance in gastronomy as a culinary herb, in perfumery, and in the pharmaceutical industry.^12^

While it has traditional uses as an emmenagogue, abortifacient, and for alleviating digestive issues, it is important to note its toxicity to both humans and animals, even at low concentrations, which makes it unsuitable for treating infestations such as fleas. Additionally, the oil is recognized for its insect-repelling properties, further highlighting its diverse applications in herbal medicine.^13^

The EO of *M. pulegium* has been the subject of numerous studies, with several chemotypes being identified. Research has revealed that its composition is predominantly composed of oxygenated monoterpenes, such as pulegone, piperitone, menthone, and menthol. However, the chemical profile of this EO can vary significantly depending on the geographical region where the plant is harvested.^14,15^ Despite its rich phytochemical profile, few studies have explored the antimicrobial, antioxidant, and insecticidal properties of *M. pulegium* EO.^12,16^

In Algeria, research on *M. pulegium* has been conducted in a few regions,^9^ yet its presence remains undocumented in the country’s national parks. To bridge this gap, this study aims to inventory *Mentha* species in two distinct regions Attatba (Tipaza) and Hammam Melouane (Blida) and compare the chemical compositions of their EOs. By identifying the active constituents and chemotypes, this research contributes to the potential valorization and broader application of *M. pulegium* EOs.

With growing concerns about antibiotic resistance and the need for environmentally friendly pest control, natural products such as *M. pulegium* EOs are gaining renewed interest. However, regional variations and a limited understanding of their mechanisms of action hinder their widespread application. Therefore, this study aimed to (1) characterize the chemical composition of *M. pulegium* EOs from Attatba and Hammam Melouane; (2) evaluate their in vitro antimicrobial, antioxidant, and insecticidal activities; and (3) elucidate potential mechanisms of action through molecular docking simulations, to better inform their practical application.

## 2 Materials and Methods

### 2.1 Plant Material

This study focused on the aerial parts (stems and leaves) of *M. pulegium* growing naturally in two distinct regions: Attatba (Tipaza, Algeria; 36°35′31″N, 2°26′58″E) and Hammam Melouane (Blida, Algeria; 36°19′12″N, 3°02′42″E). The plant material was collected in March 2023 at altitudes of 398 m in Hammam Melouane and 280 m in Attatba. Voucher specimens were prepared and authenticated by the Department of Botany at the École Nationale Supérieure d’Agronomie (ENSA) in El Harrach, Algiers, Algeria. This process was based on herbarium specimens collected and identified by the same department, under the voucher numbers 027/23 (Attatba) and 028/23 (Hammam Melouane). Approximately 12.3 kg of fresh material was collected from each region.

### 2.2 Microbial Strains

The microbial strains, provided by the SAIDAL Laboratory in Medea (Algeria), were reference strains from the American Type Culture Collection (ATCC). These strains were identified and characterized by the Pasteur Institute of Algiers (Algeria). Three strains were selected for testing: a Gram-positive bacterium (*Staphylococcus aureus* ATCC 6538), a Gram-negative bacterium (*Escherichia coli* ATCC 8739), and a yeast (*Candida albicans* ATCC 10231).

### 2.3 Animal Material

The insecticidal potential of phyto-preparations derived from the EO of *M. pulegium* aerial parts was evaluated via the use of a green citrus aphid (*Aphis spiraecola* Pach). The aphids were collected from infested clementine leaves (*Citrus reticulata* Blanco) at the experimental station of the Institut National de la Protection des Végétaux (INPV) in Boufarik (Blida, Algeria). A sample of 60 aphids was used for each trial. The pest was identified by qualified personnel from INPV Boufarik.

### 2.4 Extraction of EOs and Yield Determination

Essential oils were extracted from the fresh aerial parts of *M. pulegium* collected from Attatba and Hammam Melouane by hydrodistillation, a traditional and widely used technique for EOs extraction.^17^ In this method, the plant material was completely immersed in distilled water within a distillation still and heated for 3 h. As the mixture boiled, steam carried the volatile compounds into a condenser, where they were cooled and condensed into a distillate composed of aromatic water (hydrosol) and EOs. The EOs were subsequently separated based on differences in density.

According to Helena et al.,^18^ hydrodistillation involves immersing botanical material in boiling water, which helps protect EOs from thermal degradation. The surrounding water serves as a barrier, preventing localized overheating. This method is particularly suitable for preserving delicate volatile compounds. The EOs were collected, stored at 4 °C in amber-colored airtight vials, and protected from light until use. The EOs yield, expressed as a percentage (%), was calculated as the ratio of mass (in grams) of the EOs to the mass of the fresh plant material.

### 2.5 Chemical Composition Analysis of EOs

The chemical composition of the EOs was analyzed via a Hewlett-Packard Agilent 6800 gas chromatograph coupled with a Hewlett-Packard Agilent 5973 mass spectrometer. Prior to GC–MS analysis, the EO was diluted in *n*-hexane at a ratio of 10 μL of EO to 1 mL of *n*-hexane. Electron impact fragmentation was performed at 70 eV. The GC–MS system was equipped with an HP–5MS column (30 m × 0.25 mm, 0.25 μm film thickness) with a stationary phase of 5% phenyl and 95% dimethylpolysiloxane. The oven temperature program ranged from 45 to 280 °C at a rate of 2 °C/min. The injector temperature was set at 250 °C, and pure helium was used as the carrier gas at a flow rate of 0.5 mL/min. Injection was performed in split mode (split ratio: 1/20) with a volume of 0.2 μL. The mass spectrometer was operated in scan mode, acquiring data from *m*/*z* 50 to 500. The system was controlled by “HP ChemStation” software, and mass spectra were compared with the NIST 98 library.

### 2.6 In Vitro Antioxidant Activity

To evaluate the antioxidant activity of the EOs, two complementary methods were used: the DPPH (2,2-diphenyl-1-picrylhydrazyl) radical scavenging assay and the ABTS (2,2′-azino-bis (3-ethylbenzothiazoline-6-sulfonic acid)) radical scavenging assay.

The DPPH radical scavenging capacity was assessed following the method described by Sarikurkcu et al. (2012),^19^ with slight modifications. A series of dilutions (0.01–0.1 μg/mL) was prepared, and 50 μL of each EO was mixed with 950 μL of a 60 μM DPPH methanolic solution. After 30 min of incubation in the dark at room temperature, the absorbances were measured at 517 nm. The scavenging activity, expressed as percentage inhibition (*I* %), was calculated using eq 1.

1 $$I\;\left(\%\right)=100\frac{A_{c}-A_{e}}{A_{c}}$$

where *A*_c_ is the absorbance of the control sample and where *A*_e_ is the absorbance of the test sample.

The IC_50_ value (concentration required to scavenge 50% of the DPPH radical) was determined from the linear regression equation of the curve. Ascorbic acid served as the positive control. All experiments were performed in triplicate.

The ABTS radical scavenging activity was assessed using the method described by Obanor et al. (2013).^20^ The ABTS radical was generated by reacting ABTS (7 mM) with potassium persulfate (70 mM) in equal volumes. The mixture was kept at room temperature in the dark for 16 h to allow the formation of ABTS^•+^ cations. This solution was then diluted with methanol to obtain an initial absorbance of approximately 0.700 at 734 nm. One hundred microliters (100 μL) of each EO were added to 2 mL of the ABTS solution, and the absorbance was measured at 734 nm. The percentage of inhibition was calculated using the same equation as for the DPPH assay. All experiments were carried out in triplicate.

### 2.7 In Vitro Antimicrobial Activity

The microbial media utilized for cultivating the bacterial and fungal strains were Muller–Hinton agar and Sabouraud dextrose agar, respectively. Inocula for each strain were prepared from fresh cultures by selecting 3 to 5 well-isolated, identical colonies with a sterile loop. These colonies were suspended in physiological saline (0.9% NaCl) and thoroughly mixed by vortexing. The antimicrobial potential of *M. pulegium* EOs from Attatba and Hammam Melouane was assessed via the agar diffusion test (aromatogram), as described by Tyagi et al. (2014).^21^ The positive controls included antibiotic disks containing amoxicillin (15 μL) and a hexamidine antiseptic solution (0.1%) to evaluate the antibacterial and antifungal properties, respectively. Sterile absorbent disks (9 mm diameter) were impregnated with the EO, diluted in dimethyl sulfoxide (DMSO) at three concentrations (20, 40, and 60 μL/disk), and carefully placed in the center of Petri dishes via sterile forceps. Each test was conducted in triplicate. The Petri dishes were sealed and incubated at appropriate temperatures: 37 °C for 24 h for bacteria and 25 °C for 72 h for yeast. The microbial inhibitory effect was determined by measuring the diameter of the inhibition zone (DZI), including the 9 mm disk diameter, and comparing it with the results obtained with standard antibiotics.

### 2.8 In Vitro Insecticidal Activity via Contact Toxicity

The contact toxicity of *M. pulegium* EO formulations from two distinct regions was evaluated in the entomology laboratory of the INPV regional station in Boufarik (Blida, Algeria). All experiments were conducted in accordance with the biosafety and pesticide handling guidelines established by the INPV in Algeria. Ethical approval was not required for this study; however, appropriate safety measures were taken when handling EOs and chemical agents. Additionally, all experimental protocols were executed under controlled laboratory conditions to ensure safety and reproducibility.

During the experiment, the ambient conditions were maintained at a temperature range of 26–28 °C and a relative humidity of 60–80%. Prior to conducting the contact toxicity assay for the two EOs, several preliminary tests were performed to determine the optimal doses for evaluation. Subsequently, four doses (D_1_, D_2_, D_3_, and D_4_) were prepared by diluting 100, 150, 200, and 250 μL of each EOs in 100 mL of distilled water containing Tween 80, respectively. To assess the efficacy of the essential oil-based formulations, methoxyfenozide, an approved insecticide until March 31, 2026 (Regulation No. 1107/2009), was used as a positive control. While data on green aphids is limited, the literature suggests that concentrations ranging from 10 to 100 μmol/L (diluted in DMSO) are appropriate for in vitro testing. Therefore, four concentrations (C_1_ to C_4_) were prepared at 20, 40, 60, and 80 μmol/L. A preparation without any active ingredient (Tween 80 and distilled water) served as the negative control. Each 9 cm Petri dish was lined with Whatman No. 1 filter paper, onto which a clementine leaf infested with 20 green aphids was placed. The prepared dilutions were then sprayed onto each green aphid within each Petri dish, across all doses and controls, using a sprayer. Each assay was repeated three times.

The prepared dilutions were sprayed onto the aphids via a sprayer. Each experiment was repeated three times. Aphid mortality was assessed via a magnifying glass, with an insect considered dead if it showed no response to the touch of a slightly heated needle. Observations were recorded at 1, 3, 6, 12, and 24 h posttreatment. Mortality rates were calculated as a function of exposure time and applied dose. To account for natural mortality, the recorded mortalities were corrected via Abbott’s formula (eq 2).^22^

2 $$M_{c}\left(\%\right)=\left(\frac{M-M_{t}}{100-M_{t}}\right)\times 100$$

where *M* is the percentage of dead aphids in the treated population and where *M*_*t*_ is the percentage of dead aphids in the control population.

Additionally, lethal doses (LD_50_ and LD_90_) were determined for the EOs from both regions, representing the doses required to kill 50% and 90% of the insect population, respectively. All trials were conducted in triplicate to ensure reproducibility.

### 2.9 Statistical Analysis

The biological activities of the EOs were compared via one-way analysis of variance (ANOVA) to determine whether significant differences existed between the treatments. When ANOVA indicated significant differences (*p* < 0.05),^23^ Tukey’s honest significant difference (HSD) test was performed as a post hoc analysis to identify specific pairwise differences among the groups. All the statistical analyses were conducted via Python’s SciPy and StatsModel libraries.^23^

### 2.10 In Silico Molecular Docking

This study employed molecular docking simulations to investigate the potential interactions between selected ligands (primary compounds) and protein targets associated with antimicrobial, antioxidant, and insecticidal activities. The ligands, which include (+)-4-carene, (+)-limonene, menthone, piperitenone, piperitone, and pulegone (structures illustrated in Figure 1), were retrieved in SDF format from the PubChem database (https://pubchem.ncbi.nlm.nih.gov/) and converted to 3D Protein Data Bank (PDB) format using Discovery Studio 2024. The protein structures were obtained from the PDB for DNA gyrase (PDB ID: 1KZN), lanosterol 14-alpha demethylase (PDB ID: 5TZ1),^24^ human peroxiredoxin (PDB ID: 1HD2),^25^ and acetylcholinesterase (PDB ID: 4EY7).^26^

**Figure 1:**
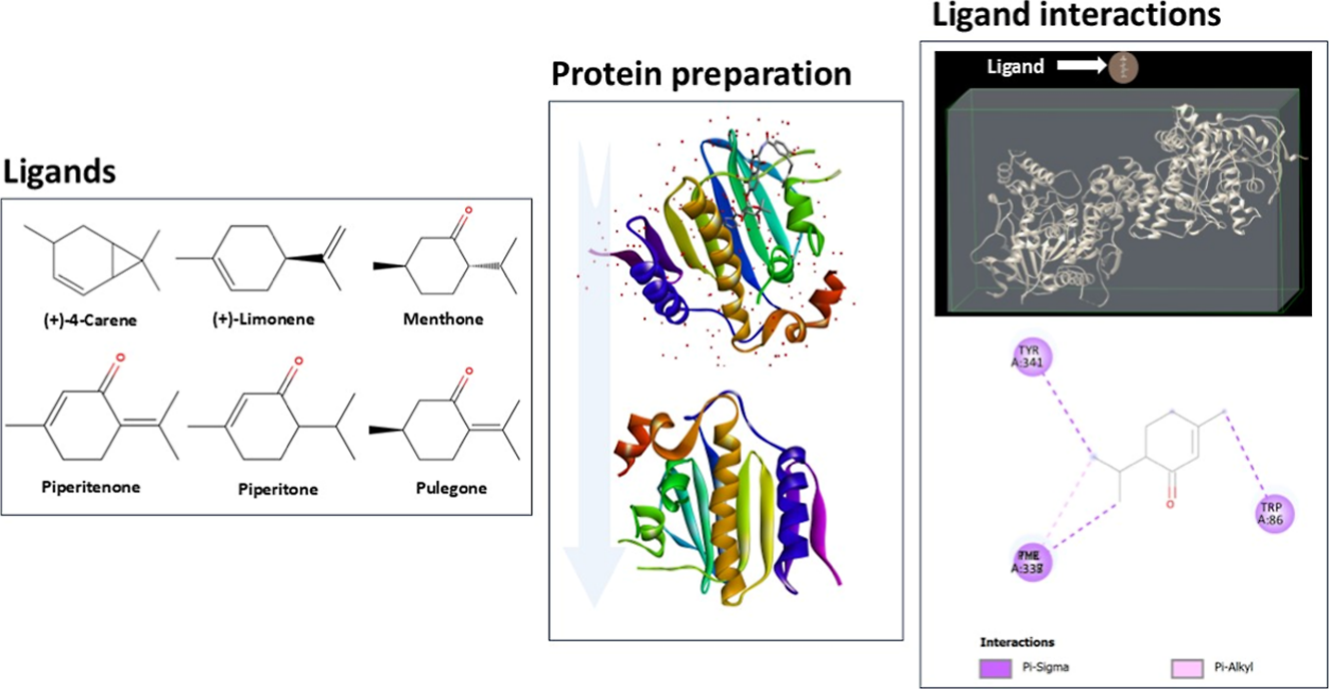
Illustration of the key steps in the molecular docking study: ligand and protein structure retrieval, preparation and optimization, and analysis of binding poses.

The protein preparation involved cleaning the downloaded structures by removing water molecules, ions, and cocrystallized ligands via Discovery Studio 2024 and polar hydrogens were added and Gasteiger charges were assigned to all atoms using AutoDock Tools.^27^ The energy minimization step was performed using the steepest descent algorithm in Chimera to relieve steric clashes. Furthermore, missing residues were added via Chimera 1.18. The protein was then converted to PDBQT format, with charges and atom types set for optimal docking performance. The ligand structures were prepared by assigning partial charges and defining rotatable bonds.

Molecular docking simulations were conducted via Chimera version 1.18. The active site was defined as a cubic box with the following dimensions.

- DNA gyrase (1KZN) is centered at coordinates 43.243 (*x*), 41.9555 (*y*), and 36.1724 (*z*).
- Human peroxiredoxin (1HD2) is located at coordinates 8.38048 (*x*), 41.2515 (*y*), and 20.1506 (*z*).
- Acetylcholinesterase (4EY7) is located at coordinates −3.95003 (*x*), −50.6316 (*y*), and 0.877182 (*z*).

The number of binding modes was set to 10 for each simulation, with a search exhaustiveness of 8, and the maximum energy difference was established at 3 kcal/mol. After docking, the results were analyzed, binding affinities were predicted, and intermolecular interactions and distance measurements were obtained via Discovery Studio 2024.^27^

## 3 Results and Discussion

### 3.1 Inventory of the *Mentha genus* in the Two Regions

Our survey, which was conducted in two regions, namely, Attatba (Tipaza, Algeria) and Hammam Melouane (Blida, Algeria), allowed us to identify three (03) species belonging to the Mentha genus in the Attatba region. These species are *Mentha spicata*, *M. pulegium*, and *Mentha rotundifolia*. In contrast, one additional species, *Mentha suaveolens*, was found in the Hammam Melouane region. Figure 2 shows the Mentha species inventoried in both study regions.

**Figure 2:**
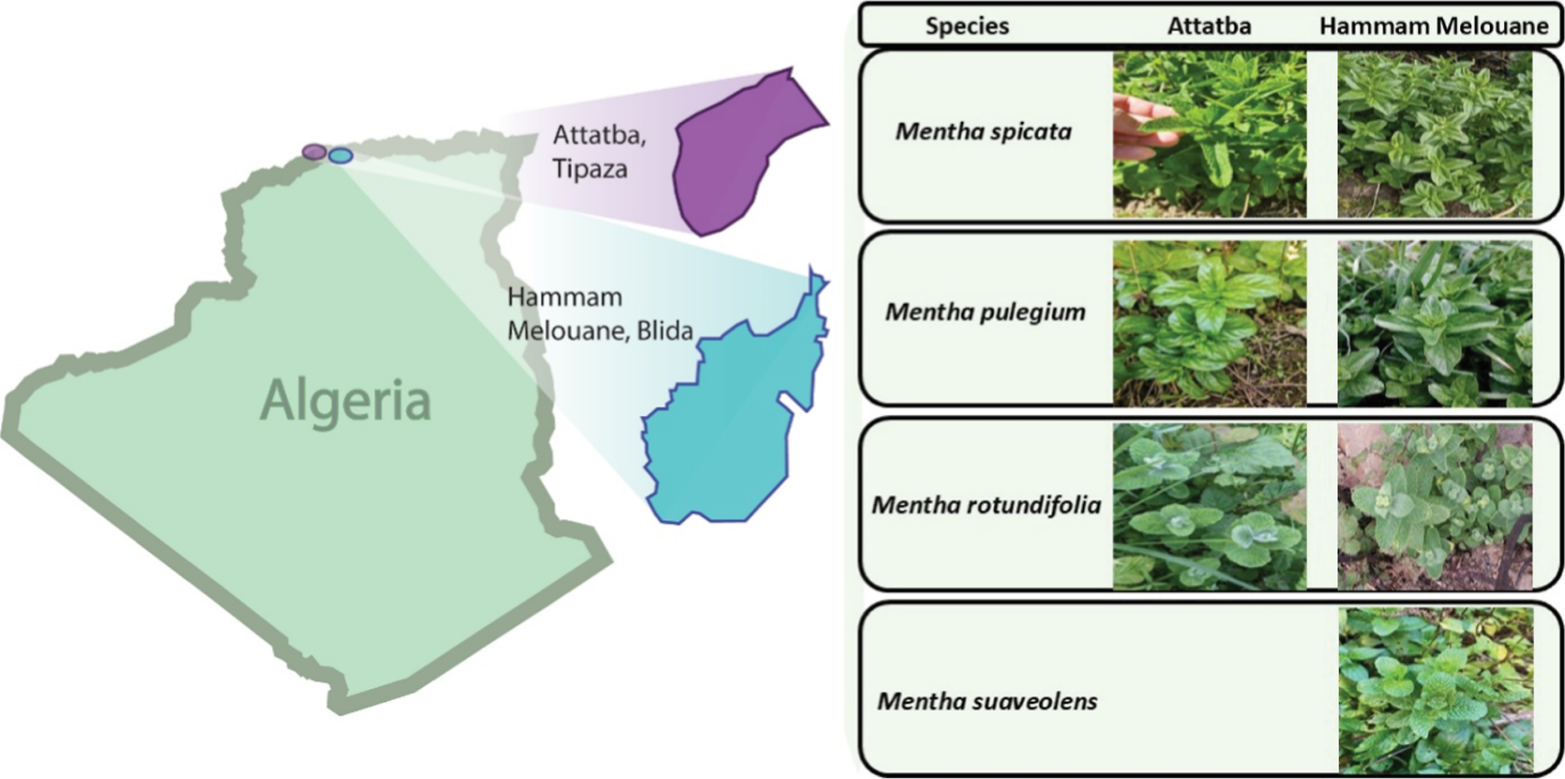
Species of the *M. genus* identified in the two regions.

The *M. genus* in Algeria presents a fascinating case study in terms of biogeography and botanical discovery. While extensive research continues to illuminate the diverse properties of mint, the composition of the Mentha flora of Algeria remains a subject of evolving understanding. Recent reports challenge previously held assumptions about the distribution and diversity of these aromatic herbs across the country. For example, Benabdallah et al. (2018) revealed the unexpected presence of six Mentha species—*Mentha aquatica*, *Mentha arvensis*, *M. x piperita*, *M. pulegium*, *M. rotundifolia*, and *M. x villosa*—within El Kala National Park, a finding that extends the known range of these species in Algeria.^15^ In addition to this complexity, Ouakouak et al. (2019) documented *Mentha citrata* in the southeastern region of El-Kobna (El Oued), further highlighting the geographic variability of the *Mentha* distribution.^28^

In contrast, Brahmi et al. (2020) identified a more limited set of *Mentha* species—*M. rotundifolia*, *M. pulegium*, *M. spicata*, *M. aquatica*, and *Mentha longifolia*—across three distinct Algerian regions.^29^ The disparity between these findings, where some inventories record species previously unconfirmed in Algeria while others suggest a smaller set of widely distributed taxa, underscores the need for continued and comprehensive botanical surveys to fully characterize the diversity and distribution of Mentha within the various landscapes of Algeria. This ongoing taxonomic exploration emphasizes that our understanding of even well-studied plant genera can be surprisingly incomplete, necessitating ongoing research to refine our knowledge of regional biodiversity.

### 3.2 Extraction Yield and Chemical Composition

The EO yields calculated from fresh material varied among samples. Hydrodistillation extraction yielded 0.5 ± 0.2% in Attatba and 0.42 ± 0.1% in Hammam Melouane. These yields are lower than those obtained in Morocco via dried material, as reported by Zekri et al. (2013) in M’rirt (5.29%), Azrou (5.9%), and Khénifra (6.2%)^30^ and by Allali et al. (2021) in the Ouazzane region (2.14%).^16^ Brahmi et al. (2016) reported an EO yield of 1.14% from dried material in the locality of Samaoun (Bejaia, Algeria).^12^ Additionally, the EO content of *M. pulegium* collected in El Kala National Park (El Tarf, Algeria) was 1.8%,^15^ whereas it was 1.45% in the Bouira region (Algeria).^31^ Other studies conducted in various parts of the world also reported varied yields. The EO yield of *M. pulegium* collected in Northwest, South, and Southwest Iran ranged from 0.3% to 1.7%,^32^ with a yield of 0.65% reported by Kamkar et al. (2010),^33^ and in Turkey, yields ranged from 0.3% to 1.2%.^34^

GC–MS analysis allowed the identification of 38 compounds from the EOs of the aerial parts (stems and leaves) of *M. pulegium* from both regions: Hammam Melouane and Attatba. These represent a total of 96.99% and 93.82% of the chemical composition, respectively. The results are presented in Table 1.

**Table 1:** Chemical Composition of EOs from the Aerial Parts of *M. pulegium* Collected in the Two Regions, as Determined via GC–MS^a^

regions/compounds	Hammam melouane (%)	Attatba (%)	RI	RI_R_
1 α-thujene	0.02	-	932	929
2 α-pinene	0.88	1.67	938	935
3 α-terpinolene	0.05	0.09	948	995
4 camphene	0.13	0.12	969	957
5 sabinene	0.08		975	975
6. β-pinene	0.67	4.07	981	981
7 myrcene	-	0.65	989	994
8. 3-octonone	0.92	1.53	993	998
9 α-terpinene	-	0.05	999	1019
10 *p*-cymene	-	0.19	1028	1026
11 (+)-limonene	3.59	**41.99**	1032	1034
12. α-ocimene	0.04	-	1036	1039
13. β-ocimene	-	0.09	1045	1043
14 *Trans*-ocimene	0.05	-	1049	1049
15. 3-octanol	1.46	1.18	1054	994
16. γ-terpinene	-	0.15	1058	1058
17. (+)-4-Carene	**7.97**	-	1064	1018
18. fenchone	-	0.07	1089	1090
19. 4-terpineol	-	0.22	1099	1148
20. myrtenal	-	0.28	1123	1187
21. camphor	0.63	-	1142	1145
22. menthone	**6.17**	-	1148	1153
23. *Cis*-*p*-menth-2,8-dienol	-	1.39	1154	1120
24. 3,4-dihydropyran	-	3.32	1159	1133
25 iso-menthone	1.63	0.22	1162	1181
26 menthol	5.18	-	1165	1170
27 pulegone	**61.00**	0.29	1167	1236
28 3-*p*-menthene	2.25	-	1176	1182
29. bornyl acetate	0.14	-	1185	1286
30. carvone	-	0.16	1193	1199
31. piperitone	-	**23.08**	1213	1228
32. seudenone	-	0.89	1228	1039
33. piperitenone	0.39	**12.06**	1334	1374
34. α-copaene	0.08	-	1345	1375
35. β-bourbonene	0.05	-	1349	1382
36. β-caryophyllene	-	0.66	1416	1420
37. α-caryophyllene	1.52	-	1437	1438
38- (+)-β-selinene	2.09	-	1474	1478
total (%)	96.99	93.82		
monoterpene hydrocarbons	13.48	49.07		
oxygenated monoterpenes	77.25	37.77		
sesquiterpene hydrocarbons	3.74	0.06		
other	2.52	6.92		

a (−) absence, RI: retention index calculated on an apolar column (HP5-MS), RI_R_: A reference retention index from literature^35–37^ and Pherobase and PubChem databases.

Chromatographic analysis of *M. pulegium* EO from Hammam Melouane revealed a composition consisting primarily of oxygenated monoterpenes, at 77.25%, with pulegone being the most abundant compound at 61%, followed by (+)-4-carene (7.97%) and menthone (6.17%). The other compounds were present at levels lower than 6%. The percentage of monoterpene hydrocarbons was 13.48%. These compounds were the most abundant in the Attatba EO, accounting for 49.07%, with (+) limonene (41.99%) being the major component, followed by piperitone (23.08%) and piperitenone (12.06%). The other compounds were present at less than 5% purity. The percentage of oxygenated monoterpenes was 37.77%. However, sesquiterpene hydrocarbons were found at low levels in both EOs, and oxygenated sesquiterpenes were practically absent in both.

*M. pulegium* EO from the Hammam Melouane region (Blida) was characterized by a pulegone chemotype. The EO from the Attatba region (Tipaza) presented a (+)-limonene/piperitone/piperitenone chemotype. These results agree with earlier studies that have shown that the chemical composition of *M. pulegium* EO consists mainly of oxygenated monoterpenes such as pulegone, piperitenone, isomenthone, and piperitone and monoterpene hydrocarbons such as limonene.^38–40^

The chemical profile of *M. pulegium* EO exhibits remarkable variability depending on geographic origin and environmental conditions. Studies conducted across Algeria consistently identify pulegone as a dominant component; however, its concentration fluctuates significantly, ranging from approximately 70% to nearly 90% in certain regions of Bejaia.^12,31,41^ This pattern extends beyond Algeria, with Moroccan *M. pulegium* EOs displaying similarly diverse compositions, often with pulegone predominating but at varying percentages.^30,42,43^ Even within Iran, the pulegone content has a broad range (2.5%–51.7%), indicating a strong influence of environmental factors on EO composition.^32^

For a broader comparison, an analysis was conducted that considers various regions across different continents, as illustrated in Figure 3.^44–50^ The analysis demonstrates that while pulegone predominates in certain areas, such as Italy (86.2%) and Uruguay (73.4%), other regions exhibit markedly different chemical profiles. For instance, the Chilean EO is noteworthy for containing comparable levels of pulegone (29.33%) and menthol (28.79%), which deviates from the pulegone-dominant oils typically reported. Similarly, the Bulgarian oil is characterized by a relatively high concentration of piperitenone, indicating a distinct regional chemotype.

**Figure 3:**
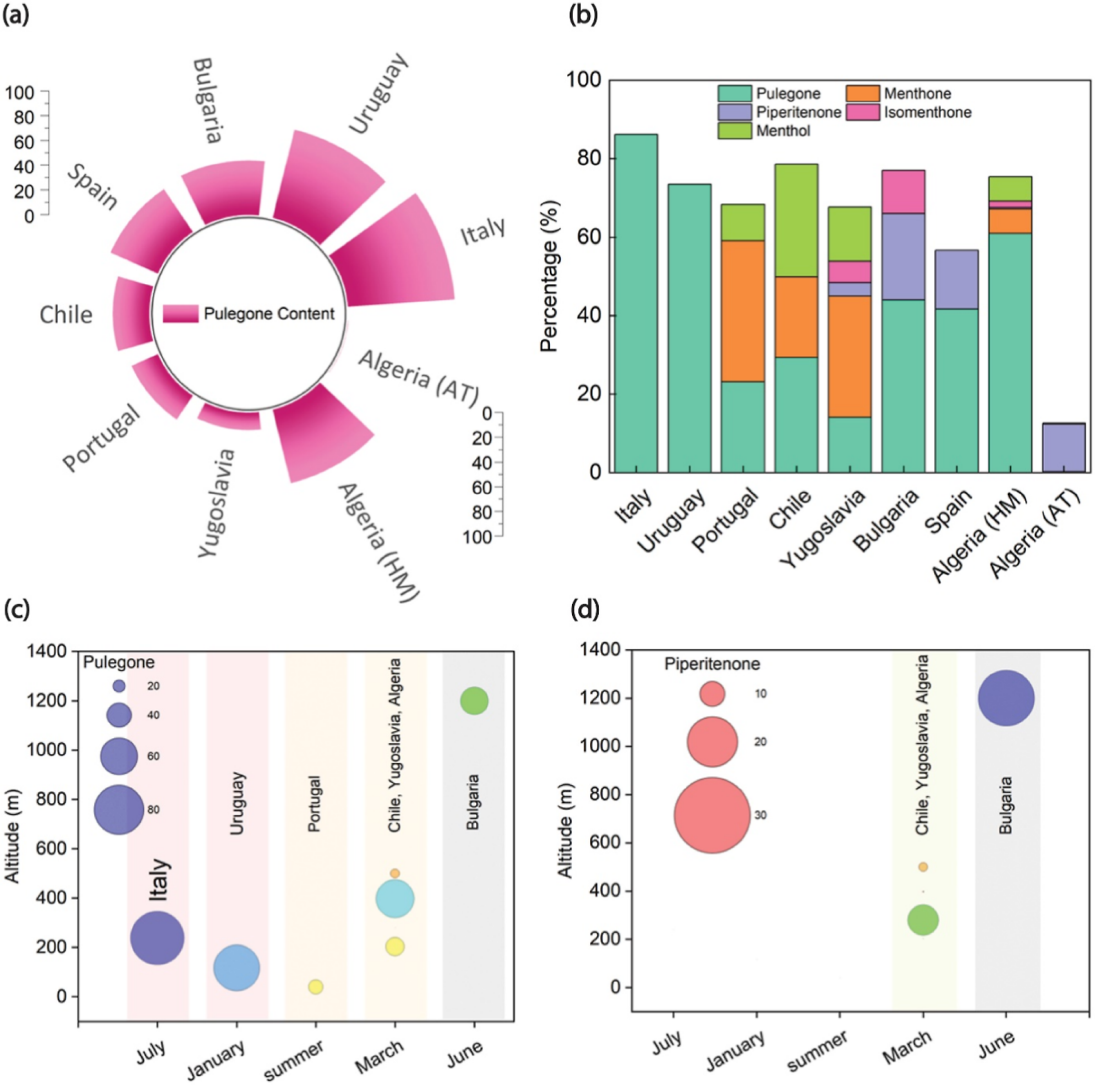
(a) Percentage of pulegone content in *M. pulegium* EO from various geographical locations. (b) Relative percentages of major compounds (pulegone, menthone, piperitenone, isomenthone, menthol) in *M. pulegium* EO from different regions. (c) Pulegone concentrations in relation to altitude and harvest time. (d) Piperitenone concentrations in relation to altitude and harvest time.

Figure 3c,d provide valuable insights into the potential influence of altitude and harvest time on the content of pulegone and piperitenone. Based on the available data for the studied regions, there are no consistent correlations of altitude or time of year with production trends. The high pulegone content of the Italian sample (July) does not conclusively indicate that higher altitudes generally promote pulegone synthesis, as *M. pulegium* EO from Uruguay (January) exhibits a nearly identical percentage of pulegone content despite differing altitude and season. However, for piperitenone and with these two specific data points, it is reasonable to suggest that its accumulation may be related to other environmental factors such as more intense solar radiation and other time of year related factors.

### 3.3 In Vitro Antioxidant Activity

The in vitro antioxidant properties of the *M. pulegium* EOs extracted from Attatba and Hammam Melouane were assessed using DPPH and ABTS radical scavenging assays, with ascorbic acid serving as a positive control (Table 2). The IC_50_ values obtained for both EOs indicate a dose-dependent antioxidant activity, where higher concentrations were required to inhibit 50% of the radicals compared to ascorbic acid. Importantly, the lower the IC_50_ value is, the greater the antioxidant activity.

**Table 2:** Antioxidant Properties of the Two *M. pulegium* EOs in Vitro^a^

samples	DPPH	ABTS
	IC_50_ (μg/mL)	IC_50_ (μg/mL)
Hammam Melouane EO	318.03 ± 2,03^b^	453.11 ± 2.15^b^
Attatba EO	536.62 ± 4,05^a^	650.89 ± 3.13^a^
ascorbic acid	5.54 ± 0,01^c^	6.37 ± 0.02^c^

a Different letters indicate statistically significant differences between samples within the same column (*p* < 0.05), as determined by Tukey’s HSD test.

Specifically, the Hammam Melouane EO exhibited a DPPH IC_50_ of 318.03 ± 2.03 μg/mL, while the Attatba EO demonstrated a higher IC_50_ value of 536.62 ± 4.05 μg/mL. Similarly, in the ABTS assay, the Hammam Melouane EO had an IC_50_ of 453.11 ± 2.15 μg/mL, and the Attatba EO had an IC_50_ of 650.89 ± 3.13 μg/mL. Ascorbic acid, a well-known antioxidant, displayed significantly lower IC_50_ values in both assays (5.54 ± 0.01 μg/mL for DPPH and 6.37 ± 0.02 μg/mL for ABTS), indicating its superior radical scavenging capacity compared to the EOs.

Statistical analysis using one-way ANOVA followed by Tukey’s HSD test confirmed significant differences in antioxidant activity among the tested samples (*p* < 0.05). The IC_50_ values indicated that Hammam Melouane EO exhibited significantly greater antioxidant potential than Attatba EO, as evidenced by their distinct statistical groupings. However, both EOs demonstrated significantly lower antioxidant activity compared to ascorbic acid, which exhibited the highest potency.

Ouakouak et al. (2015) reported that the EO of *M. pulegium* from the El Oued region of Algeria exhibited higher antioxidant activity than what was observed in our study (IC_50_ = 157 μg/mL).^51^ Conversely, Benabdallah et al. (2018) in El Kala National Park (El Tarf, Algeria) and Benahmed et al. (2019) in the Constantine region found lower antioxidant activity compared to our findings, with IC_50_ values of 997 μg/mL and 2293 ± 6.58 μg/mL, respectively.^15,52^ Furthermore, *M. pulegium* EOs from Tunisia and Greece demonstrated significant radical scavenging capacity, with respective IC_50_ values of 10 and 13.5 ± 0.5 μg/mL.^53,54^ The radical scavenging potential of *M. pulegium* EO collected from various bioclimatic zones in Iran has also shown considerable variation, with IC_50_ values ranging from 545 to 4884 μg/mL.^32^ The antioxidant activity of *M. pulegium* EO may be attributed to the presence of pulegone and menthone in its chemical composition. In light of the results obtained in this study and previous research, it can be concluded that *M. pulegium* EO is enriched in oxygenated monoterpenes (pulegone and menthone), which act synergistically as potential antioxidants. This conclusion aligns with the findings of Rached et al. (2025),^55^ who attributed the antioxidant effect of *M. pulegium* EO to the presence of phenolic compounds such as thymol, menthol, pulegone, and limonene, all of which are known for their antioxidant properties. Additionally, phenolic compounds enhance the antioxidant activity of EOs by acting as electron donors, neutralizing free radicals, and reducing oxidative stress. They exhibit strong radical scavenging properties due to their capacity to donate hydrogen atoms or electrons, thereby stabilizing free radicals.^56^ Therefore, the variation in antioxidant capacity can be attributed to the diversity of the chemical composition of the EO, the extraction method employed, the age of the plant, storage conditions, pedoclimatic factors, and environmental influences.^57^

### 3.4 In Vitro Antimicrobial Activity

The inhibitory effects of *M. pulegium* EOs harvested from two different regions, Attatba and Hammam Melouane, were tested against three microorganisms: a Gram-positive bacterium, a Gram-negative bacterium, and a yeast. The results of this test are presented in Table 3. The two *M. pulegium* EOs had a dose-dependent inhibitory effect. Both EOs were found to be active against all the tested strains. Specifically, both the Attatba and Hammam Melouane EOs inhibited the growth of two bacteria, *E. coli* and *S. aureus*, with DZI values ranging from 12.1 ± 0.49 to 13.1 ± 0.30 mm for the lowest dose (20 μL) and from 14.6 ± 0.89 to 16.1 ± 1.48 mm for the highest dose (60 μL). Notably, the DZI is proportional to the applied dose. For the fungal strain *C. albicans*, the DZI varied between 12.5 ± 0.25 and 19.4 ± 1.41 mm for the lowest dose and from 16.3 ± 1.38 to 28.9 ± 0.46 mm for the highest dose. Thus, *C. albicans* was more sensitive to the inhibitory action of both EOs. However, this sensitivity is more pronounced for the *M. pulegium* EO collected from the Hammam Melouane region, which could be partly due to its chemical composition.

**Table 3:** Results of the In Vitro Antimicrobial Activity of *M. pulegium* EOs from the Two Regions^a^

microbial strains	volume of EOs (μL/disc)	DZI of Attatba EO (Tipaza) (mm)	DZI of Hammam melouane EO (Blida) (mm)	positive control
Escherichia coli	20	12.1 ± 0.49^d^	12.2 ± 0.17^d^	Amox
	40	12.1 ± 0.50^e^	14.2 ± 0.41^e^	11.3 ± 0.40
	60	12.4 ± 0.15^f^	14.6 ± 0.89^f^	
Staphylococcus aureus	20	12.5 ± 1.49^g^	13.1 ± 0.30^g^	Amox
	40	13.4 ± 0.11^h^	15.1 ± 0.95^h^	13.3 ± 1.53
	60	16.1 ± 0.65^i^	16.1 ± 1.48^i^	
Candida albicans	20	12.5 ± 0.25^a^	19.4 ± 1.41^a^	Hexam
	40	13.6 ± 0.63^b^	23.8 ± 1.00^b^	10 ± 0.1
	60	16.3 ± 1.38^c^	28.9 ± 0.46^c^	

a DZI = Diameter of the Zone of Inhibition (mm), including the 9 mm disk diameter; Amox = Amoxicillin (15 μL), an antibiotic used as a positive control for bacterial strains; Hexam = Hexamidine (0.1%) antiseptic solution used as a positive control for the yeast. Values are means ± SDs (*n* = 3). Different letters indicate statistically significant differences between samples within the same column (*p* < 0.05), as determined by Tukey’s HSD test.

The antimicrobial potential of *M. pulegium* EO is widely acknowledged; however, its efficacy varies significantly across geographic regions and against different microbial targets, indicating a complex relationship between EO composition and biological activity. While some studies conducted in Algeria reported weak inhibitory effects,^12,41^ others demonstrated substantial activity against a variety of Gram-positive and Gram-negative bacteria, as well as *C. albicans*,^31^ reflecting findings from Iran, Tunisia, and Morocco.^16,53,58,59^ Frequently, pulegone, menthone, and neomenthol have been identified as key contributors to antibacterial effects, whereas menthone, menthol, carvone, and piperitenone may be responsible for antifungal activity. This regional variability highlights the intricate interplay between genetic factors, environmental conditions, and extraction methods, resulting in EOs with distinct chemical profiles and correspondingly diverse antimicrobial properties.

Statistical analysis of the antimicrobial activity of *M. pulegium* EOs from Attatba and Hammam Melouane revealed a clear dose-dependent inhibitory effect against all the tested microbial strains. Notably, *C. albicans* exhibited the highest sensitivity, particularly to Hammam Melouane EO, where the DZI reached 28.9 ± 0.46 mm at the highest concentration (60 μL). This value significantly surpasses the inhibition observed for *E. coli* and *S. aureus*, suggesting greater antifungal potential. The EO from Hammam Melouane consistently demonstrated stronger antimicrobial activity than the Attatba EO did, with significant differences observed at higher doses, particularly for *E. coli* (*p* < 0.05). Additionally, compared with positive controls, *M. pulegium* EO exhibited comparable or superior inhibition, especially against *C. albicans*, where it outperformed the hexamidine control. These findings highlight the potential of *M. pulegium* EO as a natural antimicrobial agent, with promising applications in the pharmaceutical and food preservation industries.

### 3.5 In Vitro Insecticidal Activity

The in vitro contact toxicity assay demonstrated that EOs derived from *M. pulegium*, sourced from Attatba and Hammam Melouane, were highly effective against *A. spiraecola*, resulting in significant mortality within 24 h. In contrast, the control group exhibited a mortality rate of only 8 ± 0.02% at the higher concentration of 80 μmol/L, highlighting the substantial difference in toxicity between the EOs and the control group. The deceased insects exhibited signs of immobilization, with their bodies adhered to the edges and surfaces of the Petri dishes. The results of the biocidal test conducted after 24 h indicated that the mortality rate ranged from 31.34 ± 1.7% to 81.04 ± 1.78% for the formulated EO of *M. pulegium* from the Attatba region, and from 45.17 ± 0.88% to 94.4 ± 1.10% for the formulated EO from the Hammam Melouane region (Figure 4).

**Figure 4:**
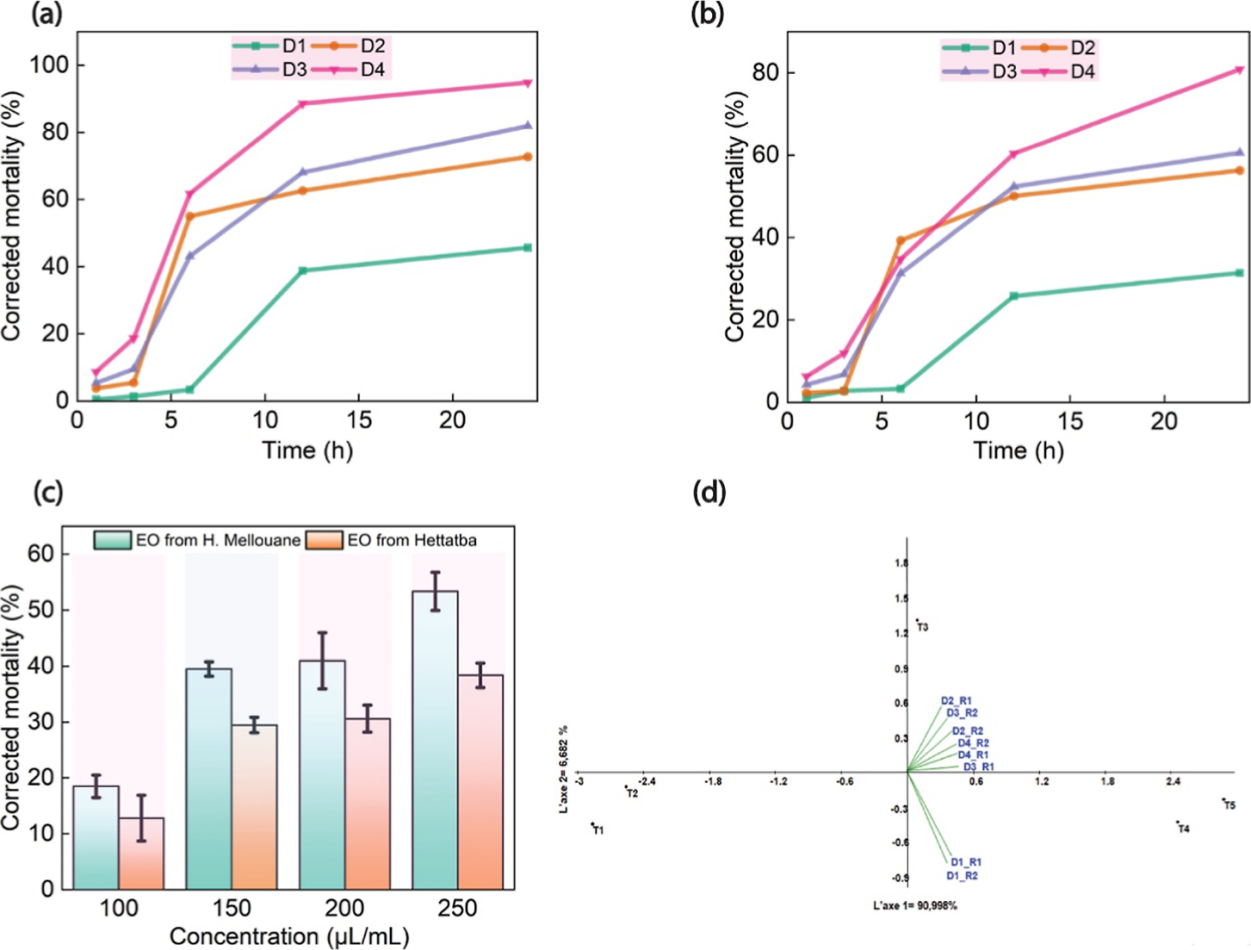
Insecticidal activity of formulated *M. pulegium* EOs against *Aphis spiraecola*. (a) Time-dependent mortality rate of *A. spiraecola* exposed to formulated EO from the Hammam Melouane region; (b) time-dependent mortality rate of *A. spiraecola* exposed to formulated EO from the Attatba region; (c) dose-dependent mortality rate of *A. spiraecola* exposed to formulated EOs from both regions after 24 h. (D_1_ = Dose 1 = 100 μL/mL, D_2_ = Dose 2 = 150 μL/mL, D_3_ = Dose 3 = 200 μL/mL, and D_4_ = Dose 4 = 250 μL/mL. The values are the means ± SDs (*n* = 3). (d) Projection of the corrected mortality of different doses and exposure times of the formulated *M. pulegium* EOs from both regions against the citrus green aphid, *Aphis spiraecola*. (R1 = Hammam Melouane, R2 = Attatba).

Methoxyfenozide exhibits selective toxicity toward larval stages, particularly those of lepidopteran pests, as extensively documented.^60^ Its mode of action accelerates the molting process, disrupting the insect’s ability to develop properly through its larval instars. However, the low mortality rate of *A. spiraecola* observed in this study indicates that aphids may be less susceptible to this synthetic insecticide, potentially due to differences in their physiological or developmental pathways compared to lepidopteran larvae. This apparent selectivity underscores the necessity of exploring alternative control strategies, such as EOs derived from *M. pulegium*, which have demonstrated a more pronounced and broad-spectrum insecticidal effect against this aphid species.

Principal component analysis (PCA), conducted via PAST software (2023), revealed that more than 96% of the variance is explained by the first two axes (Figure 4d). This test highlights the effect of each dose of the two formulated *M. pulegium* EOs from two different regions on these axes. The results of the PCA revealed that the two formulated *M. pulegium* EOs had a distinct effect on the corrected average mortality of green aphids. Specifically, the three higher doses of EOs from both regions (D2R_1_, D2R_2_, D3R_1_, D3R_2_, D4R_1_, and D4R_2_, with R1 = Hammam Melouane and R2 = Attatba) were more effective after 6 h (T3). The lower doses (D1R_1_ and D1R_2_) had efficacy after 12 and 24 h (T4 and T5). The projection of variables confirms that the dose vectors follow a trend indicating treatment effectiveness on the basis of the time of exposure and the effect of the formulated *M. pulegium* EOs from both regions on the green citrus aphid, *A. spiraecola*.

The reported insecticidal activity of formulated *M. pulegium* EO is highly variable and influenced by the source of the EO, the target pest, and the application method. While some studies, such as those on formulated EO from the Samaoun region of Algeria against *Rhizopertha dominica*, have shown minimal effects,^12^ others, such as the formulated EO from the Bouira region of Algeria tested against *Sitophilus granarius*, have achieved 100% mortality.^31^ Similarly, the *M. pulegium* EO from the Khouzestan region of Iran exhibited a significant acaricidal effect, which is corroborated by findings from different regions, such as Morocco, specifically the Ait Ourir and Ouazzane regions, demonstrating notable effectiveness against the tested pests.^16,61,62^

This activity is generally associated with the chemical composition of the EO, particularly the presence of volatile monoterpenoids that can penetrate insects rapidly.^12^ Specifically, pulegone and menthone, which are often abundant in *M. rotundifolia* EO, are recognized for their insecticidal properties.^63,64^ Furthermore, ketone and aldehyde derivatives have been demonstrated to be more toxic than alcohol and ester groups. These previous findings, in conjunction with our results, confirm the insecticidal potential of *M. pulegium* EOs from the Attatba and Hammam Melouane regions.

### 3.6 Determination of Lethal Dose LD_50_ and LD90

The lethal doses, LD_50_ and LD_90_, were determined solely for the final exposure time of 24 h. To ascertain the LD_50_ and LD_90_ of the two formulated EOs from *M. pulegium* sourced from two different regions, the corrected mortality percentages at 24 h were transformed into probabilities, while the applied doses were converted into decimal logarithms. A regression line was then plotted, depicting the relationship between the probabilities and the decimal logarithms for each EO, with the results presented in Table 4.

**Table 4:** LD_50_ and LD_90_ Values of EOs from Both Regions Applied Against *A. spiraecola* of the Clementine

EOs	LD_50_ (μL/ml)	LD_90_ (μL/ml)	slope	*R* ^2^
H. Melouane	107.6^c^	236.2^d^	2.797	0.98
Attatba	142.3^a^	394.1^b^	2.157	0.94

The table clearly demonstrates that the correlation coefficient (*R* ^2^), which measures the goodness of fit, indicates a positive correlation between the probabilities and the decimal logarithm of the doses tested for the two EOs from the respective regions. The slope, which represents the gradient of the curve, is significantly different from zero, indicating a substantial relationship between the two variables (doses and mortality rate). The *R* ^2^ values are 0.98 for the EO from Hammam Melouane and 0.94 for the EO from Attatba, suggesting that the correlation coefficients for both EOs are close to 1. This implies a strong correlation, with both variables changing in the same direction. Overall, the LD_50_ and LD_90_ values for the EOs from Hammam Melouane and Attatba were 107.6 μL/mL and 142.3 μL/mL and 236.2 μL/mL and 394.1 μL/mL, respectively.

### 3.7 Molecular Docking Results

Molecular docking plays a crucial role in the field of medicinal plants by facilitating the identification and optimization of bioactive compounds that interact with specific biological targets. This computational technique enables researchers to predict how these compounds bind to enzymes or receptors, offering insights into their potential therapeutic effects and mechanisms of action.^65^

The binding affinities of the selected ligands to the target proteins were evaluated via molecular docking simulations. The binding energies (in kcal/mol) for each ligand and protein target are summarized in Table 5.

**Table 5:** Binding Energies (kcal/mol) of Ligands to Target Proteins

ligand	binding energy (kcal/mol)		
	antimicrobial activity (ID: 1KZN)	antioxidant activity (ID: 1HD2)	insecticidal activity (ID: 4EY7)
(+)-4-Carene	–5.0	–5.2	–7.2
(+)-Limonene	–5.8	–4.7	–7.1
menthone	–5.8	–4.8	–6.9
piperitenone	**–6.4**	**–5.3**	**–7.4**
piperitone	–5.8	–5.1	–7.1
pulegone	**–6.4**	**–5.2**	**–7.2**

Molecular docking analysis revealed significant interactions between the selected ligands and three target proteins associated with antimicrobial, antioxidant, and insecticidal activities. Among all the tested compounds, piperitenone presented the most favorable binding energies across all the targets, with values of −6.4 kcal/mol for DNA gyrase (1KZN), −5.3 kcal/mol for human peroxiredoxin (1HD2), and −7.4 kcal/mol for acetylcholinesterase (4EY7). These consistently strong binding affinities suggest that piperitenone has the potential to serve as a versatile multitarget compound (Figure 5a–c).

**Figure 5:**
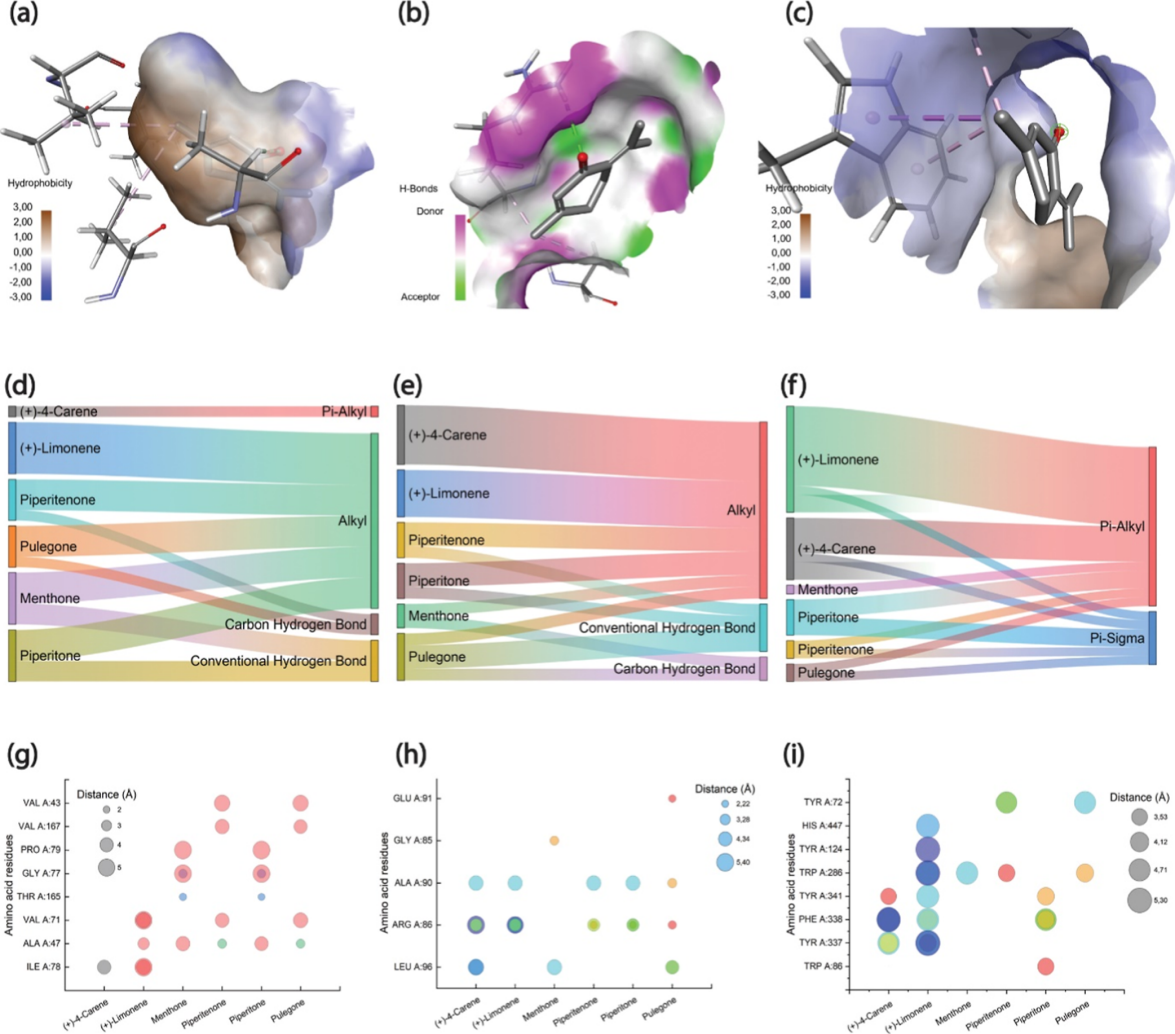
Detailed depiction of the ligand–protein interactions. (a–c) Surface interaction surface of piperitenone, which has the highest binding affinity for antimicrobial, antioxidant, and insecticide activities, respectively. (d–f) Summary of bonding modes for antimicrobial, antioxidant, and insecticide activities, respectively. (g–i) The range of amino acid residues involved in the interactions with each ligand at different distances.

For antimicrobial activity, the interactions with DNA gyrase were characterized by a combination of hydrogen bonding and hydrophobic interactions, as illustrated in Figure 5d. Piperitenone forms a crucial carbon–hydrogen bond with ALA A:47 at a distance of 2.81 Å and establishes multiple alkyl interactions with VAL residues (VAL A:71, VAL A:167, and VAL A:43) at distances ranging from 4.32 to 4.87 Å. Analysis of the binding pocket reveals a predominantly hydrophobic environment, as illustrated in the surface representation, which facilitates these interactions. Similar interaction patterns were observed for other ligands, particularly pulegone, which matched the binding energy of piperitenone and exhibited comparable interaction profiles.

With respect to antioxidant activity (Figure 5e), the docking results with human peroxiredoxin indicated that hydrogen bonding played a crucial role in ligand binding. Piperitenone forms a conventional hydrogen bond with ARG A:86 at a distance of 2.38 Å, which is supplemented by alkyl interactions with ALA A:90 and ARG A:86. The characteristics of the binding site, including distinct H-bond donor and acceptor regions, contributed to the stability of these interactions. Distance analysis revealed consistent interaction patterns across all the ligands, with most amino acid residues engaging at distances ranging from 3 to 4 Å.

The insecticidal activity (Figure 5f), assessed through interactions with acetylcholinesterase, demonstrated a distinct preference for π-system interactions. Piperitenone exhibited the strongest binding affinity in this category, with a binding energy of −7.4 kcal/mol. It formed a π-sigma interaction with tryptophan (TRP) at position A:286, measured at 3.67 Å, and a π-alkyl interaction with tyrosine (TYR) at position A:72, measured at 4.53 Å (Figure 5g–i). The abundance of aromatic amino acid residues in the binding pocket, including TRP, TYR, and PHE, facilitated these π-system interactions. Other ligands also demonstrated favorable binding energies ranging from −6.9 to −7.2 kcal/mol, indicating that this class of compounds holds significant promise for insecticidal applications.

The comprehensive analysis of binding modes and interaction types across all three proteins revealed the structural features that contribute to effective binding. The ability of these ligands, particularly piperitenone, to engage in diverse interaction types—including hydrogen bonding, hydrophobic interactions, and π-system interactions—appears to be crucial for their multitarget activity. This versatility in interaction patterns, combined with favorable binding energies, suggests that these compounds, especially piperitenone, could serve as promising leads for the development of multifunctional agents with antimicrobial, antioxidant, and insecticidal properties.

Previous research supports the insecticidal potential of piperitenone, particularly through studies investigating the effects of *Cymbopogon schoenanthus* EO and its purified component, piperitone, on the cowpea weevil, *Callosobruchus maculatus*. Ketoh et al. (2006)^66^ demonstrated that piperitone, a major constituent of *C. schoenanthus* EO, exhibits significant toxicity to *C. maculatus* adults, newly laid eggs, and neonate larvae. The purified compound displayed strong ovicidal activity, inhibited larval penetration into seeds, and caused considerable mortality among both adult weevils and developing larvae. These findings underscore the potential of piperitenone, particularly at higher concentrations, as a naturally derived insecticide for postharvest pest control. Their analysis indicated that this approach was most effective in inhibiting the penetration of neonate larvae into the seeds, utilizing steam and fractional distillation followed by flash chromatography. The results suggest that this method could be accessible for exploitation in the field.

The significance of compound interactions in enhancing biological activity is clearly demonstrated by the synergistic effects observed in this study and others (Farhanghi et al., 2022). Although each compound exhibits some antibacterial activity on its own, their combined application markedly increases biological efficacy and inflicts considerably greater damage to cell membranes than any individual treatment alone.^67^ Furthermore, the molecular docking simulations identified pulegone as a significant contributor to binding affinities. In vitro tests indicate that, to some extent, these in silico findings align with the in vitro activities observed in this study, particularly regarding the Hammam Melouane EO, which is characterized by its higher pulegone content. However, directly correlating the specific binding energies of piperitenone or any individual compound to a single in vitro assay result is challenging. The overall bioactivity of the EO likely arises from synergistic or antagonistic interactions among multiple components rather than the action of a single compound. Therefore, while molecular docking provides valuable insights into potential interaction mechanisms, further in vitro studies that specifically evaluate the individual contributions of piperitenone and pulegone to antimicrobial and insecticidal effects are necessary to fully validate the observed bioactivity trends.

## 4 Conclusions

This study comprehensively investigated the potential of *M. pulegium* EOs from two distinct regions in Algeria, Attatba and Hammam Melouane, as sources of bioactive compounds with antimicrobial, antioxidant, and insecticidal properties. By employing a combination of botanical surveys, chemical characterization, biological activity assays, and molecular docking simulations, a multifaceted understanding of the composition, activity, and underlying mechanisms of EOs can be obtained.

The botanical inventory revealed the presence of three *Mentha* species (*M. spicata*, *M. pulegium*, and *M. rotundifolia*) in Attatba, whereas Hammam Melouane processed the same three species in addition to *M. suaveolens*. GC–MS analysis further revealed distinct chemotypes, with the EO from Hammam Melouane exhibiting a pulegone-dominant profile (61.00%) and the EO from Attatba displaying a (+)-limonene/piperitone/piperitenone chemotype (41.99%, 23.08%, and 12.06%, respectively). These compositional differences correlated with variations in biological activity, as the Hammam Melouane EO generally demonstrated superior antioxidant activity. The AAI values ranged from 0.12 ± 0.02 to 0.07 ± 0.01, indicating that both exhibited weak antioxidant activity. Additionally, both EOs displayed a dose-dependent antimicrobial effect against the tested bacteria and fungi.

In insecticidal assays, both formulated EOs of *M. pulegium* effectively induced mortality in *A. spiraecola*, with mortality rates ranging from 31.34 ± 1.7% to 81.04 ± 1.78% for the Attatba EO and from 45.17 ± 0.88% to 94.4 ± 1.10% for the Hammam Melouane EO at the highest concentrations. The lethal dose (LD_50_) against *A. spiraecola* was determined to be 107.6 μL/mL for Hammam Melouane and 142.3 μL/mL for Attatba.

Molecular docking simulations identified piperitenone as a promising multitarget compound because of its capacity to interact with DNA gyrase, human peroxiredoxin, and acetylcholinesterase. The results of these experiments revealed that piperitenone has a high binding affinity of −6.4 kcal/mol for DNA gyrase (1KZN), −5.3 kcal/mol for human peroxiredoxin (1HD2), and −7.4 kcal/mol for acetylcholinesterase (4EY7), indicating significant binding potential. The intermolecular complex primarily exhibited conventional hydrogen bonds, alkyl interactions, and pi–sigma interactions.

This study provides compelling evidence that EOs derived from *M. pulegium*, specifically from the Algerian locales of Attatba and Hammam Melouane, represent a valuable resource for bioactive compounds with demonstrated antimicrobial, antioxidant, and insecticidal properties. Through a rigorous approach, the distinct chemotypes present in each region were characterized, and the specific EO with the highest affinity and selectivity toward various targets, which demonstrated strong binding capabilities, was identified. These findings unequivocally underscore the potential of this traditionally utilized plant as a source of multitarget bioactive compounds. Furthermore, they highlight the importance of *M. pulegium* EO in the discovery of new potential drugs and its relevance for future applications.

## Data Availability

All data supporting the findings of this study are included within the manuscript.
